# Controlled growth of Si-based heterostructure nanowires and their structural and electrical properties

**DOI:** 10.1186/s11671-015-0980-6

**Published:** 2015-06-23

**Authors:** Guanghan Qian, Saadah Abdul Rahman, Boon Tong Goh

**Affiliations:** Low Dimensional Materials Research Centre (LDMRC), Department of Physics, Faculty of Science, University of Malaya, 50603 Kuala Lumpur, Malaysia

**Keywords:** Heterostructure, Core-shell nanowires, Hot-wire chemical vapor deposition, NiSi, Heterojunction characteristic

## Abstract

Ni-catalyzed Si-based heterostructure nanowires grown on crystal Si substrates by hot-wire chemical vapor deposition (HWCVD) were studied. The nanowires which included NiSi nanowires, NiSi/Si core-shell nanowires, and NiSi/SiC core-shell nanowires were grown by varying the filament temperature *T*_f_ from 1150 to 1850 °C. At a *T*_f_ of 1450 °C, the heterostructure nanowires were formed by crystalline NiSi and crystalline Si that were attributed to the core and shell of the nanowires, respectively. The morphology of the nanowires showed significant changes with the increase in the filament temperature to 1850 °C. Moreover, the effect of hydrogen heat transfer from the filament temperature demonstrated significant phase changes from NiSi to Ni_2_Si with increase in the filament temperature. The increased filament temperature also enhanced reactions in the gas phase thus generating more SiC clusters and consequently formed the NiSi/SiC heterostructure core-shell nanowires at *T*_f_ of 1850 °C. This paper discusses the role of filament temperatures on the growth and constituted phase change of the nanowires as well as their electrical characteristics.

**Electronic supplementary material:**

The online version of this article (doi:10.1186/s11671-015-0980-6) contains supplementary material, which is available to authorized users.

## Background

Semiconductor nanowires have been extensively investigated due to their potential applications in a wide range of optical and electrical applications [1–3]. The hybrid heterostructures such as core-shell nanowires tend to improve the properties of the nanowires in the application of high-temperature sensing [4] and high-performance field-effect transistors [5] and the enhancement of hydrogen generation efficiency in visible photocatalytic processes [6]. SiC nanostructures are well known for their superior mechanical properties, high thermal conductivity, low thermal-expansion coefficient, good thermal-shock resistance, chemical stability, and electron affinity which make them excellent candidates for work in harsh environments [7, 8]. The incorporation of highly metallic properties of single-crystalline NiSi nanowires as core electrodes into the NiSi/SiC core-shell is expected to enhance their electrical and optical properties. The NiSi core electrodes could be used as 1D electrodes for enhancing the efficiency of electron transfer between the current collector supports and individual electrode materials as well as of ion transport to the electrode [9, 10]. Moreover, this metallic/semiconductor heterostructure could possibly enhance the carrier mobility which would significantly improve the performance of the existing nanowire-based devices [11].

Extensive work has been conducted to investigate the properties of Si-based heterostructure by various deposition techniques including chemical vapor deposition (CVD), hot-wire chemical vapor deposition (HWCVD), lithography, and laser ablation [12–15]. In this work, the Si-based heterostructure nanowires were grown by HWCVD at different filament temperatures. HWCVD is a preferred technique for the fabrication of Si-based nanowires due to its lower production costs and large-area deposition [16, 17]. Moreover, HWCVD can generate high densities of growth precursors (SiH_3_ and CH_3_) through the use of high-temperature tungsten filaments as catalyzers. In HWCVD, the filament temperature plays an important role in controlling the decomposition of the source gases and the gas phase reactions during the deposition. Since the decomposition of the SiH_4_ and CH_4_ are at different filament temperatures, the SiH_4_ starts to decompose at 1027 °C and the decomposition rate increases at temperatures above 1427 °C [18]. However, moderate decomposition of CH_4_ only occurs at 1750 °C and above [19, 20]. Therefore, HWCVD utilizes the growth of Si-based nanostructures with different morphologies and compositions by tuning the decomposition rate of the source gases by varying the hot filament temperature. This study examines the role of the filament temperature on the growth of different morphologies of the nanowires by HWCVD. Furthermore, we investigate the structural and electrical properties of the different morphologies of the as-grown Si heterostructure nanowires. These physical and chemical properties of the nanowires such as morphological, microstructure, and compositions are characterized by field emission scanning electron microscopy (FESEM), high-resolution transmission electron microscopy (HRTEM), X-ray diffraction (XRD) spectroscopy, and micro-Raman scattering spectroscopy. The electrical properties of the nanowires were analyzed by current–voltage (*I*–*V*) measurement.

## Methods

The nanowires were grown on SiO_2_-coated p-type crystal Si (100) substrates using a home-built HWCVD system (Additional file 1: Figure S1). The SiO_2_ was approximately 100 nm thick and its layer used to prevent the diffusion of Ni into the c-Si substrate. The deposition conditions of HWCVD for growing the nanowires are described in details elsewhere [21]. The crystal Si (100) substrates were cleaned by following RCA-I and II cleaning procedures before introduction into the reaction chamber [22]. For the RCA-II, the acid hydrofluoric cleaning procedure was avoided to prevent the etching of the SiO_2_ layer on the substrate. The Ni films, with a measured thickness of approximately 30 nm, were deposited on the heating substrates under a vacuum environment and subsequently treated by H_2_ plasma for 10 min at the pressure, plasma power, and hydrogen flow rate of 0.75 mbar, 5 W, and 100 sccm, respectively, to remove contaminations and activate the Ni surface [23]. The Ni films formed Ni nanoparticles on the surface after being treated by hydrogen plasma at a substrate temperature of 450 °C (Additional file 1: Figure S2). During the deposition, the substrate temperature and deposition pressure were fixed at 450 °C and 3 mbar, respectively, while the filament-to-substrate distance was set at 2 cm. The SiH_4_, CH_4_, and H_2_ flow rates were fixed at 1, 2, and 100 sccm, respectively, while the filament temperature was varied from 1150 to 1850 °C. The deposition time was set at 5 min.

The FESEM secondary electron images of the nanowires were obtained using a Hitachi SU 8000 SEM at a low electron-accelerating voltage of 2 kV and a working distance of 8 mm. A cross-section view of the backscattered electron images of the nanowires was collected by a Bruker photodiode-backscattered electron (PDBSE) detector. The TEM and HRTEM images of the nanowires were obtained using a TEM (JEOL JEM-2100F) with an accelerating voltage of 200 kV. The dispersing nanowires for TEM measurement were prepared on carbon-coated copper grids (Lacey 300 mesh Cu). The energy-dispersive X-ray spectroscopy (EDS) elemental mappings of the nanowire were performed using STEM/high-angle annular dark-field (HAADF) and Oxford EDS detectors. An XRD pattern was recorded over the 2*θ* range of 20 to 80° at a fixed grazing incidence angle of 1.5° using a PANalytical Empyrean X-ray diffractometer with an X-ray wavelength of 1.5406 Å. The step time and step size of the scanning were fixed at 2 s and 0.026°, respectively. The Raman spectra of the films were recorded using an InVia Raman microscope with a charge-coupled device detector and grating of 2400 lines/mm using an argon-ion laser with an excitation wavelength and laser power of 514 nm and 1 mW, respectively. A very low laser power was selected to prevent the heating effect of the laser which under normal circumstances could induce a crystallization of the Si structure [24]. The *I*–*V* measurement of the nanowires was obtained using a Keithley Source Measure Unit 236 (Keithley Instruments, Inc.) with electrical probe station (Signatone H-100). Prior to this, drops of silver paste that were used as electrodes for the *I*–*V* measurement were placed on the nanowires and the NiSi layer near their edge with an approximately 5-mm distance between the two consecutive electrodes. The details of the measurement and electrode configuration are illustrated in Additional file 1: Figure S3.

## Results and Discussion

**Fig. 1:**
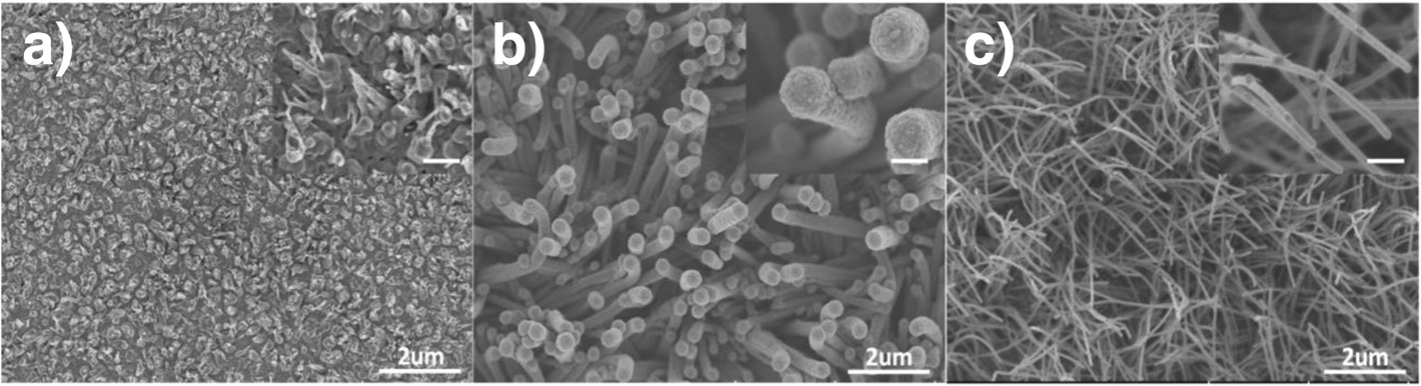
FESEM images of the nanowires prepared by HWCVD at different filament temperatures. **a**, **b**, **c** The filament temperatures of 1150, 1450, and 1850 °C, respectively. The *scale bar in the insets* is about 300 nm

The different surface morphologies of Si-based nanowires prepared by HWCVD at different filament temperatures on SiO_2_-coated crystal Si substrates are shown in Fig. 1. At 1150 °C, short NiSi nanowires, with an estimated average diameter of 20 nm, grew on top of conical-like solid particles (as shown in the inset) and became uniformly distributed on the SiO_2_ surface (Fig. 1a). They would be the bud for the core nanowire in growing the core-shell nanowires at high filament temperatures (Fig. 1b, c). At a low filament temperature of 1150 °C, there is almost no decomposition of SiH_4_, CH_4_, and H_2_ by hot filament. Therefore, the impinging of these molecules on the surface of Ni nanoparticles at a substrate temperature of 450 °C was catalytically decomposed by Ni nanoparticles [25]. The decomposed Si-rich species diffuse into the Ni nanoparticles forming NiSi nanoparticles and subsequently caused precipitation of the NiSi nanowire buds. The growth of these NiSi nanowires follow the nucleation limited silicide reaction which has been described by Kim et al. [26]. The increase in the filament temperature to 1450 °C and the high density of rod-like NiSi/Si nanowires is presented in Fig. 1b with the latter demonstrating a grainy surface morphology as seen in the inset. The estimated average diameter and length of these nanowires are typically 400 nm and 2 μm, respectively. At temperatures exceeding 1450 °C, the hot filament is able to efficiently decompose the SiH_4_ and H_2_ leading to the creation of high-density Si-rich species onto the surface of Ni nanoparticles [18]. This increases the diffusion of Si-rich species into the Ni nanoparticles thus enhancing the formation of NiSi and the subsequent precipitation of the NiSi nanowires. The excess Si-rich species would be deposited later onto the NiSi nanoparticle surface and precipitate radially following the NiSi nanowires as a shell of the nanowires. A further increase in filament temperatures up to 1850 °C shows a significant decrease in diameter to an average of 70 nm and an increase in the length to 4 μm of the nanowires. As presented by Dasgupta et al. [27], filament temperatures above 1850 °C allow sufficient decomposition of SiH_4_, H_2_, and CH_4_ molecules. In this condition, an additional diffusion of the C-rich species forms a SiC layer on the surface of the NiSi nanoparticles which could prevent the further diffusion of Si-rich species. The formation of SiC as a shell layer can limit the radial growth of Si nanocolumns as reported in our previous works [28, 29]. Therefore, these nanowires show whisker-like nanowires with small diameters as compared to those grown at 1450 °C. Further, it is worth noting that the stems of the nanowires showed formations of branched nanoneedles which seem to grow on the NiSi particles on the sidewalls of the nanowires as demonstrated in the inset. The formation of the NiSi nanoparticles could be due to the out-diffusion of NiSi from the core of the nanowires. The out-diffusion of the metal catalysts during the growth of nanowires has also been observed by Hannon et al. [30]. The estimated average tip radius of the sharp nanoneedles is about 5 nm, and such hierarchical ultra-sharp nanoneedles on the sidewalls can provide an extremely strong local field effect in field emission applications.

**Fig. 2:**
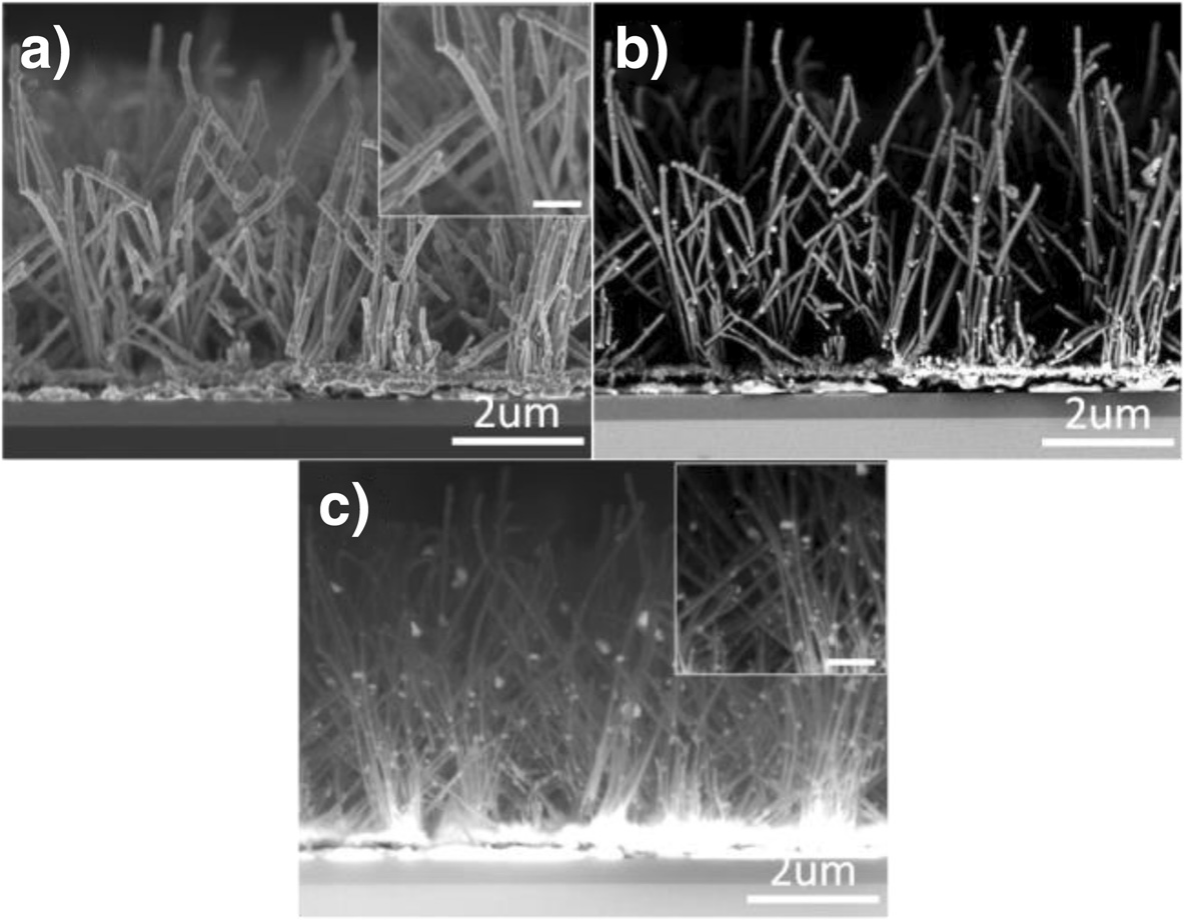
Cross-section FESEM images of the nanowires prepared by HWCVD at filament temperature of 1850 °C. **a**, **b** The FESEM images in the secondary electron and backscattered electron signals, respectively. **c** The backscattered electron signal collected by a photodiode-backscattered electron (PDBSE) detector attached to the SEM. *Insets* of the figures present high magnification of the FESEM images. The *scale bar in the insets* is about 1 μm

The cross-section FESEM images of typical nanowires which were prepared at filament temperatures above 1850 °C provided more detailed information including that on surface topography, compositions, and internal structure of the nanowires. Figure 2a depicts a secondary electron image that clearly shows the nanowires grown on top of the NiSi/SiO_2_/c-Si. The use of the SiO_2_ barrier layer is to prevent the diffusion of Ni into the c-Si substrate when it is heated to above 350 °C. The estimated thickness of the NiSi layer is about 140 nm, and this increased thickness is due to the diffusion of Si and C into the Ni nanoparticles thus forming the NiSi layer. This NiSi subsequently catalyzed into the growth of the NiSi/Si and NiSi/SiC core-shell nanowires at filament temperatures of 1450 and 1850 °C, respectively. As can be seen from the figure, the nanowires grow vertically although their alignment is not uniform when they are longer. In addition, the surface of the nanowires is smooth and less grainy as compared to the NiSi/Si nanowires grown at 1450 °C (see inset). The backscattered electron image of the cross-section nanowires is illustrated in Fig. 2b and shows the presence of bright particles sticking to their surface. Generally, the BSE image is used to illustrate the distribution of heavier elements presented on the surface of the nanostructures, and the high density of metals provides a significant contrast compared to the matrix in the BSE image. Apparently, the bright particles on the surface of the nanowires reveal the presence of NiSi nanoparticles which were found randomly distributed on the surface along the nanowires. Their formation could be due to the out-diffusion of NiSi from the core of the nanowires during the growth processes. These NiSi nanoparticles are the solid catalysts that induce the growth of the hierarchical ultra-sharp nanoneedles on the sidewalls of the nanowires as mentioned above.

Figure 2c demonstrates a backscattered electron signal of the nanowires detected by an external photodiode-backscattered electron (PDBSE) detector. By applying a higher electron accelerating voltage of 15 kV, the internal structure of the nanowires can be clearly observed through the image (see inset). Furthermore, the distribution of the compositions can also be illustrated by the PDBSE image which clearly reveals the heterostructure core-shell nanowires where the core and shell could be attributed to the NiSi and SiC, respectively. Moreover, the NiSi core can be clearly presented along the nanowires from the root to the tip. The formation of the NiSi nanoparticles is also clearly presented on the surface along the nanowires. On the other hand, these nanowires which grow on top of the NiSi layer and the core nanowires are strongly connected to the NiSi layer and could form a good electrical contact for energy storage applications such as lithium-ion batteries and micro-supercapacitor electrodes. The large surface area of the nanowires is expected to enhance the performance of these devices.

**Fig. 3:**
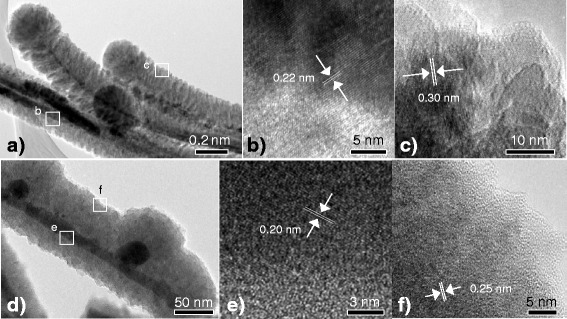
TEM and HRTEM images of the nanowires prepared by HWCVD at filament temperatures of **a**–**c** 1450 and **d**–**f** 1850 °C. The arrows in the figure indicate the measuring of a single lattice fringe

The TEM and HRTEM images of the nanowires prepared at filament temperatures of 1450 and 1850 °C are shown in Fig. 3 and demonstrate a clear core-shell structure as shown in Fig. 3a, d. At 1450 °C, the nanowires exhibit a hierarchically flower-like structure with a core diameter and shell thickness of approximately 10 and 130 nm, respectively. The shell branches are consistent along the length of the nanowires. The HRTEM image near the edge of the core reveals a single crystalline structure of the nanowire as shown in Fig. 3b. The estimated lattice spacing was approximately 0.22 nm, corresponding to a crystalline Ni_3_Si_2_ (210) crystallographic plane [JCPDS card No. 00-024-0524]. Figure 3c shows a HRTEM image at the sidewall of the shell revealing a polycrystalline structure of nanocolumns. The estimated lattice spacing is approximately 0.30 nm and corresponds to a crystalline Si (111) crystallographic plane [JCPDS card No. 00-001-0787]. This polycrystalline Si formed the nanocolumns of the hierarchical shell branches of the nanowires with their estimated diameter ranging from 20 to 24 nm. The nanowires turn to a whisker-like structure with the increase in the filament temperature to 1850 °C with thinner shell structure. The estimated core diameter and shell thickness were approximately 10 and 30 nm, respectively. It is worth mentioning that the diameter of the core was unchanged for the two filament temperatures implying that they have no effect on the diameter of the NiSi core nanowires. Accordingly, the substrate temperature is used to initiate the growth of the NiSi core as observed in Fig. 1a. The filament temperature actually acts to enhance the growth of the core and the shell of the nanowires through the introduction of growth precursors to the growth sites and the hydrogen-assisted heat transfer effect. Further, the formation of the NiSi nanoparticles on the core and, consequently, the out-diffusion of the NiSi from the core can be clearly observed in the figure. These phenomena support the formation of the branch nanoneedles at the stem of the nanowires. The NiSi core shows a single crystalline structure with a lattice spacing of 0.20 nm corresponding to Ni_2_Si (202) crystallographic plane [JCPDS card No. 00-065-1507], as depicted in Fig. 3e. Also, the shell of the nanowire exhibits an amorphous structure with the presence of embedded nanocrystallites within an amorphous matrix (Fig. 3f). The estimated lattice was approximately 0.25 nm, corresponding to SiC (111) crystallographic plane [JCPDS card No. 00-002-1050]. The elemental maps of the NiSi core and SiC shell are presented in Additional file 1: Figure S4.

**Fig. 4:**
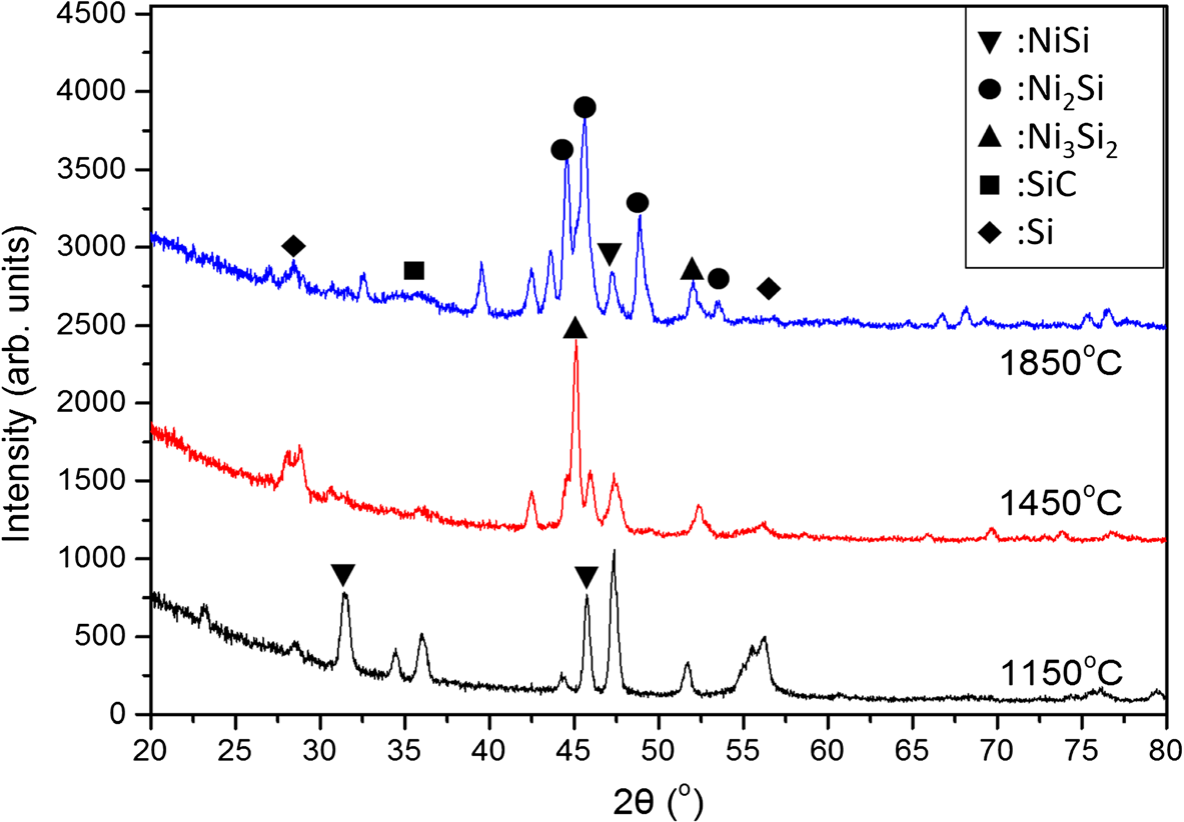
XRD spectra of the nanowires prepared by HWCVD at different filament temperatures

Figure 4 shows the XRD patterns of the nanowires prepared at different filament temperatures. The samples mainly demonstrate the presence of three major phases of crystalline nickel silicide such as NiSi, Ni_2_Si, and Ni_3_Si_2_. The crystalline NiSi with an orientation of (211) plane located at a diffraction angle of 45.6° is seen to be dominant among the phases indicating that the NiSi core could be in a (211) growth direction. Instead of NiSi, other phases could form the NiSi nanoparticles present on the surface along the nanowires, and since their formation is random, it results in the formation of different crystalline planes. Furthermore, crystalline Si peaks were observed at diffraction angles of 28.4 and 56.3° which correspond to the crystalline Si (111) and (311) planes, respectively. At the highest filament temperature of 1850 °C, a small and broad peak was detected at the diffraction angle of 35.7° which is attributed to the crystalline 3C-SiC with a crystalline orientation of the (111) plane. The crystalline size of the SiC nanocrystallites can be estimated by using Scherrer’s equation as $D=\frac{k\lambda }{\beta \cos \theta }$, where *k*, *λ*, *β*, and *θ* are the Scherrer’s constant (0.9), the wavelength of X-ray (1.5089 Å), and the full-width half-maximum of the diffraction peak and Bragg angle of the diffraction peak, respectively [31]. The average crystallite size of the SiC nanocrystallites is about 3 nm. By rough estimation from the PDBSE image, the diameter of the core and shell is about 7 and 70 nm, respectively, showing the presence of SiC nanocrystallites most probably embedded within an amorphous matrix in the shell of the nanowires.

**Fig. 5:**
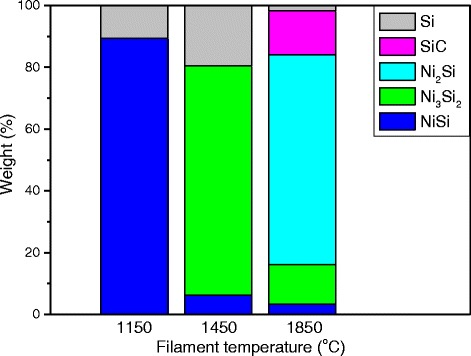
Weight percentage of phases presented in the samples prepared by HWCVD at different filament temperatures

The percentage of phases presented in the samples prepared at different filament temperatures are shown in Fig. 5 with their amounts determined by obtaining their areas using Gaussian fitting method. At the lowest filament temperature of 1150 °C, the sample mainly consisted of the NiSi phase with a little amount of crystalline Si phase while there is no decomposition of SiH_4_ and CH_4_ by hot filament. The arrival of SiH_4_ molecules on the Ni nanoparticles was catalytically decomposed by the Ni nanoparticles although that usually occurred at very low SiH_4_ decomposition rates. Therefore, only small amounts of the crystalline Si phase were observed, and the formation of the NiSi phase is most dominant through the diffusion of Si into the Ni nanoparticles. With the increase in the filament temperature to 1450 °C, most of the NiSi phase converted to Ni_3_Si_2_ while the percentage of the crystalline Si phase increases. This could be the reason for the growth of NiSi/Si core-shell nanowires which are catalyzed mainly by the Ni_3_Si_2_ phase. With a further increase in the filament temperature to 1850 °C, the Ni_3_Si_2_ phase decreased with the increase of the Ni_2_Si and SiC phases. The conversion of Ni_3_Si_2_ to Ni_2_Si may be due to an increase in the surface temperature attributed to the hydrogen-assisted heat transfer from the hot filament at the temperature of 1850 °C (Additional file 1: Figure S5). Further, a high decomposition rate of CH_4_ leads to the diffusion of the C-rich species into the NiSi nanoparticles which subsequently form a radial SiC layer. The formed Ni_2_Si and SiC phases eventually precipitated into the Ni_2_Si core and SiC shell of the heterostructure nanowires. The rise in the surface temperature increases the solubility of C into the NiSi nanoparticles thus relatively reducing the amount of Si in the NiSi phase (50 % of Si in NiSi at 1150 °C, 40 % of Si in Ni_3_Si_2_ at 1450 °C, and 33 % of Si in Ni_2_Si at 1850 °C). This eventually results in the thin and long whisker-like nanowires caused by the formation of the SiC shell layer which limited the radial growth of the Si nanocolumns.

**Fig. 6:**
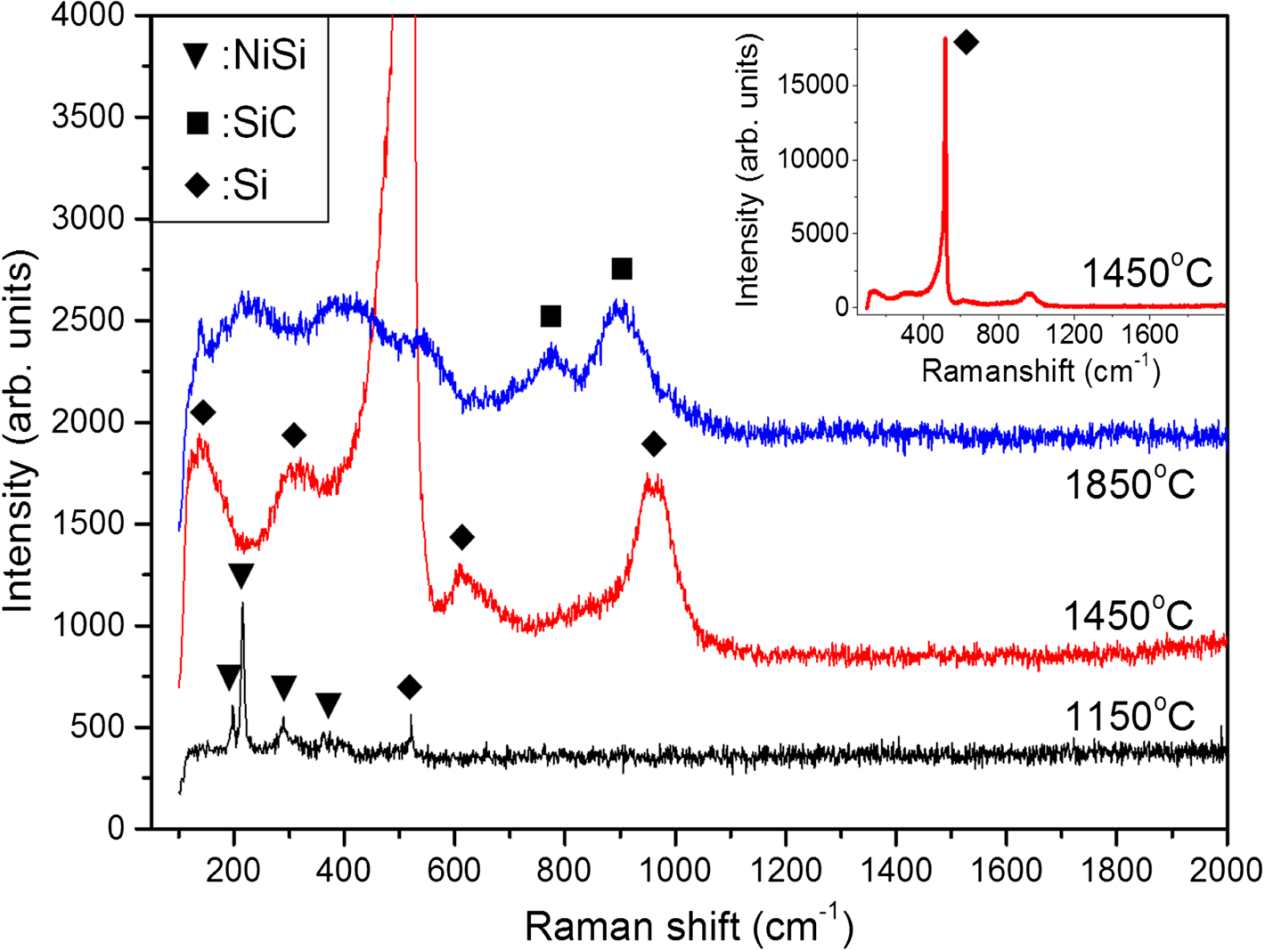
Raman scattering spectra of the nanowires prepared by HWCVD at different filament temperatures. *Inset* of the figure shows full spectrum of Raman scattering spectrum of the nanowires prepared at filament temperature of 1450 °C

The Raman scattering spectra of the nanowires prepared at different filament temperatures are shown in Fig. 6 and consist mainly of NiSi, Si, and SiC excitation bands. Typical Raman excitation bands of NiSi are depicted by Raman spectrum for the sample prepared at a filament temperature of 1150 °C while those of the NiSi phases are located at 196, 213, 295, and 367 cm^−1^. A small crystalline Si transverse optical (TO) band appeared at 520 cm^−1^ indicating the presence of small amounts of the Si phase in the sample which matches the low catalytic decomposition of SiH_4_ by the Ni nanoparticles. The filament temperature of 1450 °C produced a sharp crystalline Si TO band at 520 cm^−1^ as shown in the inset. Other Si phases are also clearly observed at 150, 300, 620, and 960 cm^−1^ and are associated with transverse acoustic (TA), longitudinal acoustic (LA), longitudinal optical (LO), Si-H vibration, and second order of Si TO (2TO) modes, respectively [32]. The dominant phase of the crystalline Si component accompanying the small amounts of amorphous component implies the formation of the polycrystalline Si shell. The growth of these heterostructure single crystalline NiSi/polycrystalline Si core-shell nanowires has been reported previously [33]. At the highest filament temperature of 1850 °C, the Raman scattering spectrum clearly demonstrated the appearance of SiC peaks at 780 and 910 cm^−1^ corresponding to the TO and LO SiC excitation modes, respectively. This reveals the formation of 3C-SiC nanocrystallites embedded within an amorphous matrix in the SiC shell, as illustrated in Fig. 3f.

**Fig. 7:**
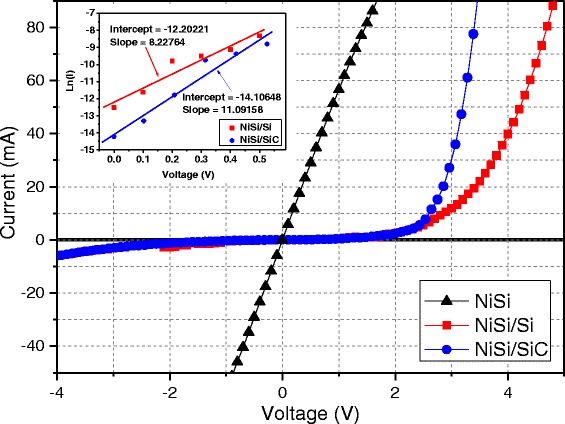
*I*–*V* curves of the samples prepared by HWCVD at different filament temperatures. *Inset* of the figure presents the logarithmic current against voltage (ln*I*–*V*) plot of the NiSi/Si and NiSi/SiC core-shell nanowires prepared at filament temperatures of 1450 and 1850 °C, respectively

Figure 7 shows the *I*–*V* curves of the different types of Si-based heterostructure nanowires. The schematic diagram of the cross-sectional view for the fabricated Si-based nanowire heterojunction is shown in Additional file 1: Figure S3. For the *I*–*V* measurement, the structure was covered with silver paste over an effective area of about 0.785 mm^2^. *I*–*V* curves of the Si-based nanowires at different filament temperatures are demonstrated in Fig. 7. At the filament temperature of 1150 °C, NiSi nanowires showed a linear *I*–*V* curve indicating a significant metallic characteristic with an ohmic contact between them and the Ag electrode. The estimated resistivity is larger than that of a single crystalline NiSi nanowire which was previously reported at about 9–20 μΩ cm. The high value of resistivity could be due to the measurements conducted on the large area of the nanowires which involves resistance from the deposited NiSi layer and the series of resistances occurring between the electrodes. On the other hand, the *I*–*V* curves clearly demonstrated a rectifying current flow in the variation of voltage for the Si-based heterostructure core-shell nanowires prepared at filament temperatures of 1450 and 1850 °C. This indicates a diode characteristic of the nanowires which could have originated from the contact between the metallic NiSi core and the semiconducting Si and SiC shells for the nanowires prepared at 1450 and 1850 °C, respectively. For a heterojunction diode based on the assumption that the current is due to thermionic emissions, the relation between the applied forward bias and current can be expressed as [34]

1 $$ I={I}_o\left\{ \exp \left[\frac{q\left(V-I{R}_{\mathrm{s}}\right)}{nkT}\right]-1\right\}+\left(V-I{R}_{\mathrm{s}}\right){G}_{\mathrm{p}} $$

where *n* is the ideality factor, *I*_*o*_ is the reverse saturation current, *T* is the temperature in Kelvin, *q* the electronic charge, and *k* is the Boltzmann constant. *G*_p_ and *R*_s_ are the parallel parasitic conductances and series resistance, respectively, which are important parameters influencing the electrical characteristic of a diode. At very small voltage regions, the *n* was obtained from slope of the ln*I*–*V* plot as shown in the inset,

2 $$ n=\frac{q}{kT}\left(\frac{\partial V}{\partial \ln I}\right) $$

Compared to the ordinary diode, the Si-based heterojunction diodes exhibited a relatively larger ideality factor. This could be due to a combination of metal–semiconductor junctions and other heterojunctions in series with each node having a corresponding ideality factor. The ideality factor of Si-based heterojunction diode is equal to the superposition of all [35] as described below:

3 $$ n={\displaystyle \sum_i{n}_i} $$

*I*_*o*_, *R*_s_, and *G*_p_ are extracted by the fitting of the measured *I*–*V* curve following Eq. (1). The results of the fitting for the NiSi/Si and NiSi/SiC core-shell nanowires (see Table 1) show smaller series resistance compared to those which could be related to the different morphological effects of the nanowires.

**Table 1:** The experimental values of the parameters obtained from I–V fittings

Type of diode	Ideality factor	Reverse saturation current (A/cm^2^)	Series resistance (Ω)	Parallel conductances (1/Ω)
NiSi/Si	4.701	1.583E−4	6.132	0
NiSi/SiC	3.487	6.125E−8	4.772	8.306E−4

## Conclusions

Different types of Si-based heterostructure nanowires were grown by HWCVD at different filament temperatures, and their morphological, structural, and electrical properties are presented in this study. The filament temperature was a major factor in controlling the decomposition of the source gases (SiH_4_ and CH_4_) and gas phase reactions that led to the growth of different types of the nanowires. The growth of core NiSi nanowires followed the nucleation limit silicide reaction while the formation of crystalline Si and amorphous SiC shell at 1450 and 1850 °C, respectively, were attributed to surface diffusion of the growth precursors. The formation of the crystalline Si and amorphous SiC shell were dependent on the decomposition of SiH_4_ and CH_4_ at different filament temperatures. The increased filament temperature resulted in phase changes from NiSi to Ni_2_Si due to the increased surface temperature attributed to the effect of the hydrogen heat transfer from the filament temperature. Both NiSi/Si and NiSi/SiC heterostructure core-shell nanowires exhibited heterojunction electrical characteristics which could be used for nanoscale diode applications.

## Additional file

Additional file 1: Figure S1. (a) Real picture and (b) schematic diagram of our home-built HWCVD system. Figure S2 FESEM image of the Ni film after being treated by hydrogen plasma. Figure S3 (a) A schematic diagram of the fabricated Si-based nanowires heterojunction structure and the electrodes configuration, (b) The real picture of the electrical probe measurement on the nanowires samples. Figure S4 (a) Dark-field STEM image of NiSi/SiC core-shell nanowire prepared by HWCVD at filament temperature of 1850 °C. (b) EDS elemental profile of a single nanowire at the stem. (c-f) EDS element maps of the core-shell nanowire. Figure S5 Variation of substrate temperature with filament temperature during the growth of the nanowires. T_s_, T_g_ and T_b_ represent the measured substrate temperature on the bottom surface of glass substrate, the measured temperature on the top surface of glass substrate, and the measured temperature on the top surface of crystal Si substrate, respectively.
