# Supplementary material for: Controlled growth of Si-based heterostructure nanowires and their structural and electrical properties
Source: Nanoscale Res Lett. 2015 Jun 23;10:267. doi: 10.1186/s11671-015-0980-6 (PMC4477003; doi:10.1186/s11671-015-0980-6)
Supplement: Additional file 1: Figure S1. — (a) Real picture and (b) schematic diagram of our home-built HWCVD system. Figure S2 FESEM image of the Ni film after being treated by hydrogen plasma. Figure S3 (a) A schematic diagram of the fabricated Si-based nanowires heterojunction structure and the electrodes configuration, (b) The real picture of the electrical probe measurement on the nanowires samples. Figure S4 (a) Dark-field STEM image of NiSi/SiC core-shell nanowire prepared by HWCVD at filament temperature of 1850 °C. (b) EDS elemental profile of a single nanowire at the stem. (c-f) EDS element maps of the core-shell nanowire. Figure S5 Variation of substrate temperature with filament temperature during the growth of the nanowires. Ts, Tg and Tb represent the measured substrate temperature on the bottom surface of glass substrate, the measured temperature on the top surface of glass substrate, and the measured temperature on the top surface of crystal Si substrate, respectively. [file 11671_2015_980_MOESM1_ESM.docx]

Supplementary Information for

**Controlled growth of Si based heterostructure nanowires, and its structural and electrical properties**

Guanghan Qian, Saadah Abdul Rahman, Boon Tong Goh^*^

Low Dimensional Materials Research Centre, Department of Physics, Faculty of Science, University of Malaya, 50603 Kuala Lumpur, Malaysia.

^*^Corresponding author: boontong77@yahoo.com


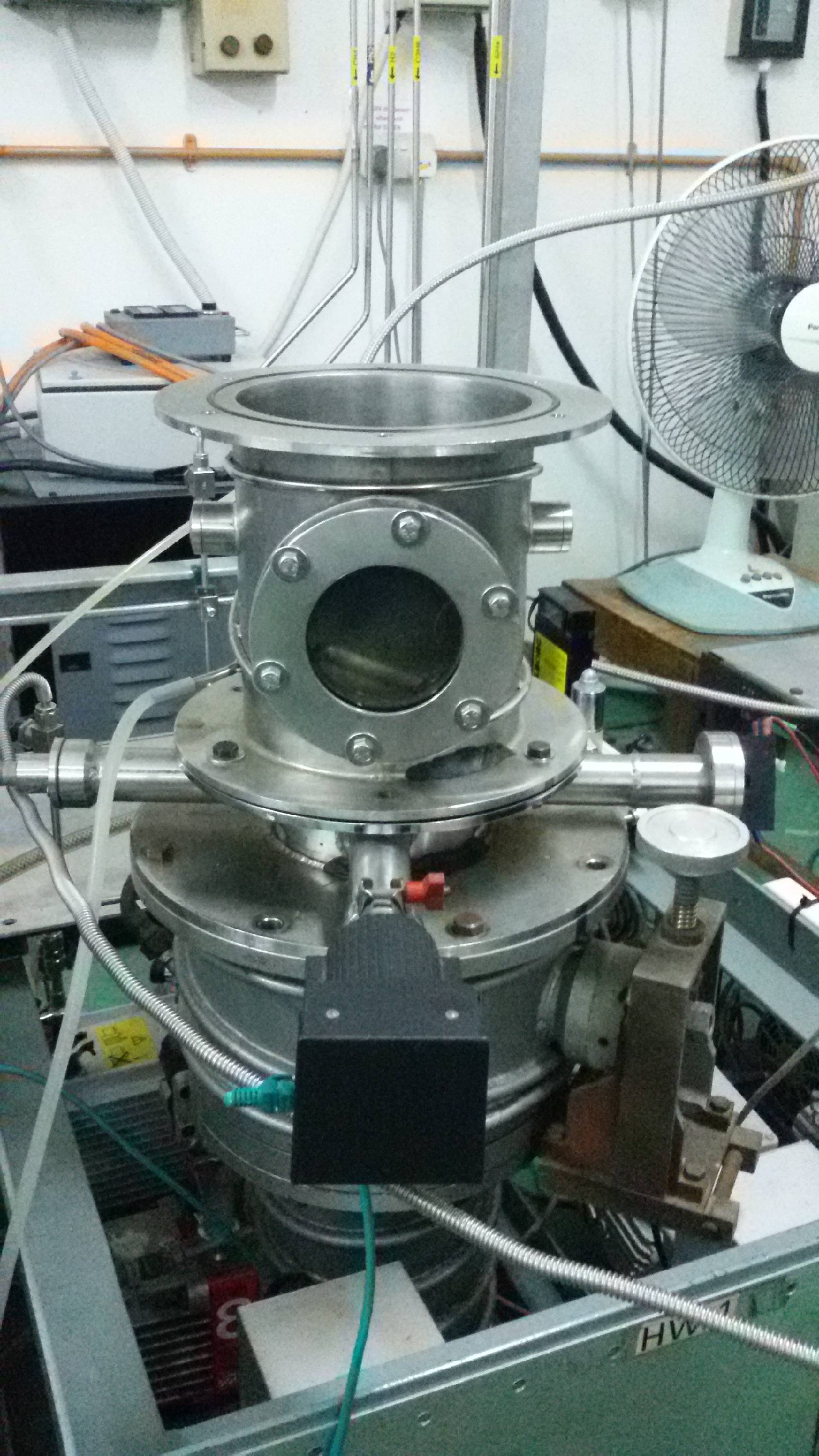


(b)

(a)

Figure S1 (a) Real picture and (b) schematic diagram of our home-built HWCVD system.


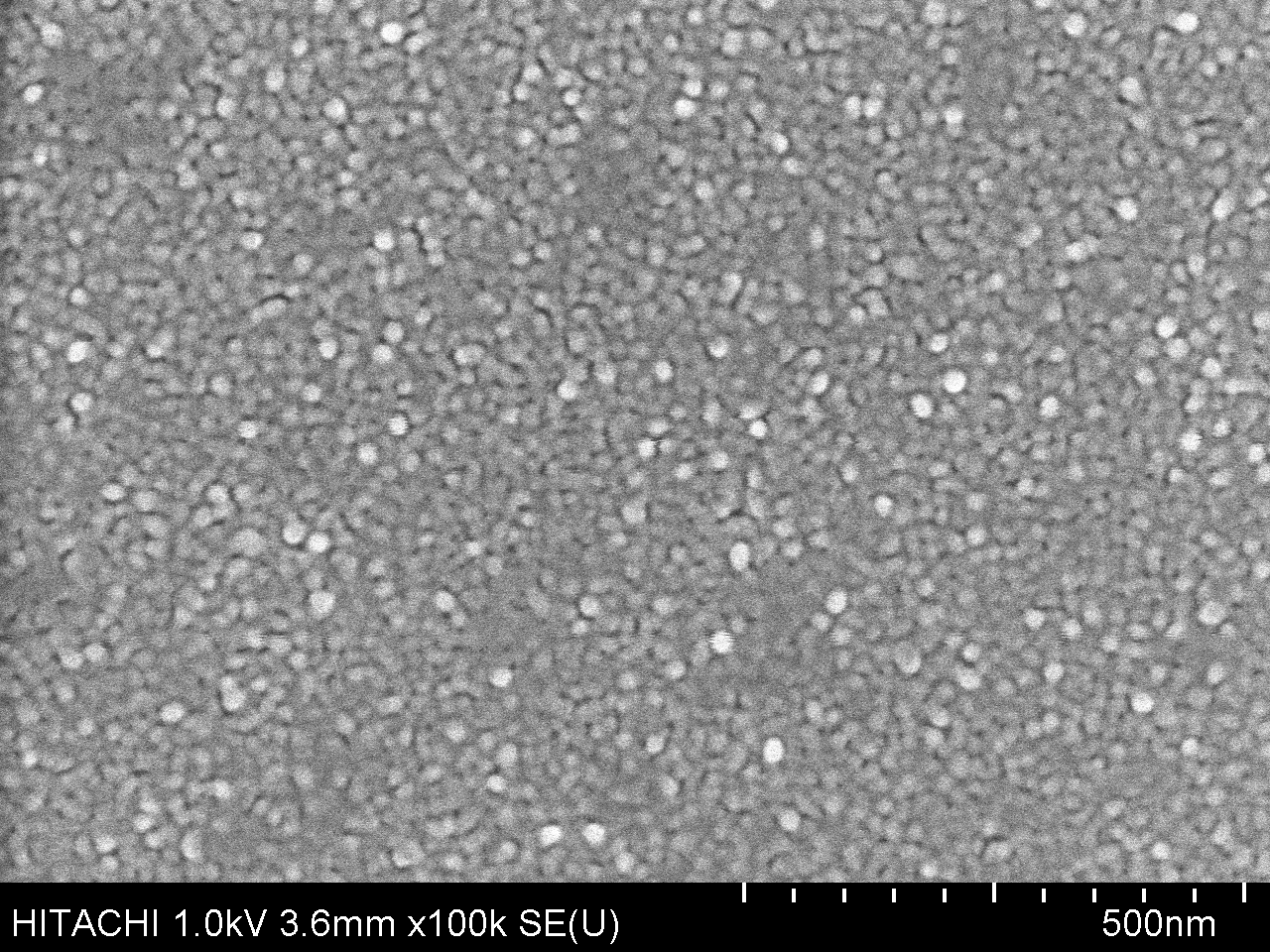


Figure S2 FESEM image of the Ni film after being treated by hydrogen plasma.


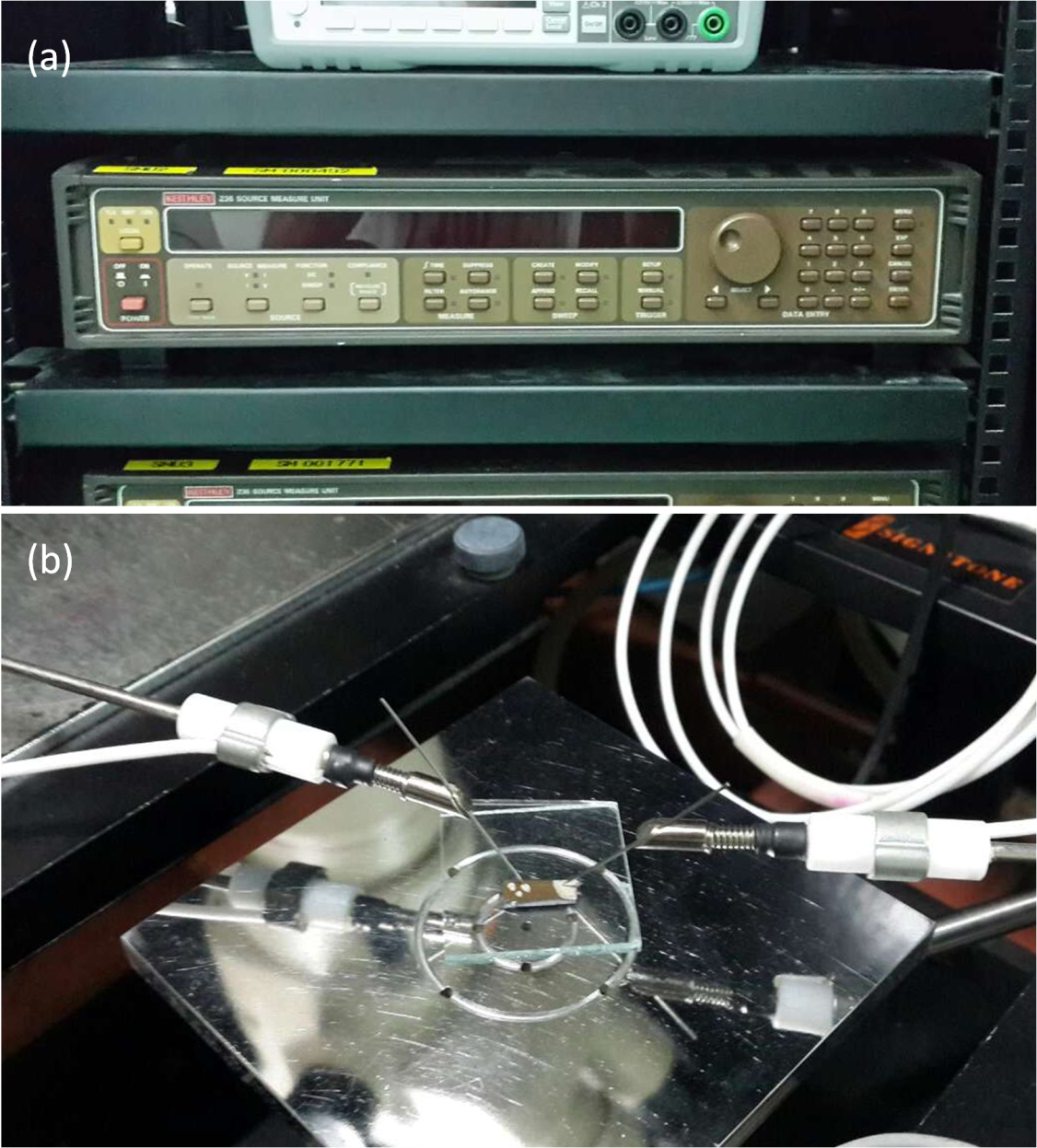


Figure S3 (a) A schematic diagram of the fabricated Si based nanowires heterojunction structure and the electrodes configuration, (b) The real picture of the electrical probe measurement on the nanowires samples.

Figure S4 (a) Dark-field STEM image of NiSi/SiC core-shell nanowire prepared by HWCVD at filament temperature of 1850°C. (b) EDS elemental profile of a single nanowire at the stem. (c-f) EDS element maps of the core-shell nanowire.

Figure S5 Variation of substrate temperature with filament temperature during the growth of the nanowires. T_s_, T_g_ and T_b_ represent the measured substrate temperature on the bottom surface of glass substrate, the measured temperature on the top surface of glass substrate, and the measured temperature on the top surface of crystal Si substrate, respectively.
